# ECG Signal as Robust and Reliable Biometric Marker: Datasets and Algorithms Comparison

**DOI:** 10.3390/s19102350

**Published:** 2019-05-22

**Authors:** Mariusz Pelc, Yuriy Khoma, Volodymyr Khoma

**Affiliations:** 1 Faculty of Electrical Engineering, Automatic Control and Informatics, Opole University of Technology, ul. Proszkowska 76, 45-758 Opole, Poland; v.khoma@po.opole.pl; 2 School of Computing and Mathematical Sciences, University of Greenwich, Park Row, London SE10 9LS, UK; 3 Department of Information Measurement Technologies, Lviv Polytechnic National University, 79013 Lviv, Ukraine; yurii.v.khoma@lpnu.ua

**Keywords:** human identification, biomarker, ECG, machine learning, Physionet, Lviv Biometric Dataset

## Abstract

In this paper, the possibility of using the ECG signal as an unequivocal biometric marker for authentication and identification purposes has been presented. Furthermore, since the ECG signal was acquired from 4 sources using different measurement equipment, electrodes positioning and number of patients as well as the duration of the ECG record acquisition, we have additionally provided an estimation of the extent of information available in the ECG record. To provide a more objective assessment of the credibility of the identification method, some selected machine learning algorithms were used in two combinations: with and without compression. The results that we have obtained confirm that the ECG signal can be acclaimed as a valid biometric marker that is very robust to hardware variations, noise and artifacts presence, that is stable over time and that is scalable across quite a solid (~100) number of users. Our experiments indicate that the most promising algorithms for ECG identification are LDA, KNN and MLP algorithms. Moreover, our results show that PCA compression, used as part of data preprocessing, does not only bring any noticeable benefits but in some cases might even reduce accuracy.

## 1. Introduction

Biometrics is a technology that is widely used as means of access control in different application domains, from smartphones and automobiles, to healthcare, e-commerce, security and the military. The main idea behind it is to perform the operation of matching a given value of a biomarker with a reference value that represents an individual [1,2].

Typically, biomarkers are some physiological and/or behavioural attributes that are unique for each human being. The most recent biometric systems rely on various biomarkers, but the most commonly used ones are based on fingerprint scanning, face and/or voice recognition, iris scanning, hand geometry, finger vein, etc.

The main requirements that a biomarker should fulfill are the following [1]:

- Universal (present for all individuals)
- Stability over time
- Easy to measure/acquire
- Low sensitivity to other physiological factors (e.g., stress, fatigue)
- Unique for each person

There are a few additional requirements that are considered a big plus:

- Fraud resistance (difficult to fake)
- Continuous nature (always available to measure)
- Liveness indication (present only from live humans)

As was shown in [3,4,5,6], the electrocardiogram signal (ECG) is a very promising biometric marker. Historically, ECG was mostly used for medical (diagnostic) purposes, but recent progress in the fields of consumer electronics and information technologies has already enabled it applications in biometric systems [7,8,9].

On the other hand, ECG-based identification systems are still not quite widespread in commercial and government services, and many of them are provided as research prototypes or very new commercial products that have just appeared on the market [10,11].

Given this situation, some questions remain open, for example ECG signal reliability and reproducibility over time, its behavior in real-world applications, the potential impact of the measurement process, hardware (sensors) configuration and matching algorithms on the identification performance, etc. 

Consequently, the main aim of this paper is to answer some of these questions and provide some estimates and insights on how robust and reliable the ECG biometric markers are. In order to accomplish this task and figure out some common trends and limitations for the ECG-based identification, we carried out some experiments for a broad range of system configurations (various datasets, matching algorithms, records length, lead type, sampling rate, ADC resolution, etc.).

## 2. Biometric System Architecture

The ECG identification process typically consists of three main stages: data acquisition, data processing (filtering, normalization, and feature extraction) and pattern matching (classification) [4,7,8,12,13].

Data acquisition requires an analogue front-end (a two- or three-electrode measurement circuit based typically on instrumental amplifiers) followed by ADC. The digitized data are then being streamed to MCU/PC. 

For the ECG measurement, we decided to choose a third-party e-Health Sensor Platform V2.0 which is based on ATmega328P. The data acquisition was performed using a differential OpAmp schema followed by an 8-bit ADC operating at a 277 Hz sampling rate [14].

The data processing includes filtering (low-pass to remove offsets and respiration, high-pass to remove noise and 50 Hz coupling, movement artifacts), heartbeats segmentation and normalization. To split the ECG waveform into separate heartbeats, it was necessary to detect the R peaks. There are multiple algorithms that have been developed for this specific purpose, but in our case a third-party implementation of the Hamilton algorithm was chosen, being available in the bioscipy library. After segmentation, each heartbeat was normalized to a range of [−1; 1]. Afterwards, the data points on the ends of each heartbeat were omitted, so only the points within the central part (around 60% of the entire heartbeat) were further used in the identification process.

Another important transformation at the data processing stage is the outlier correction. It is expected that the ECG signal is of a regular nature and that the beats tend to be similar to one another. However, for some of the beats, some strong deviations were observed. There might be different kinds of reasons for this to happen, e.g., muscle noise, respiration, non-stable contact impedance, electrodes displacement, etc. In order to detect and correct those corrupted segments (outliers), a special algorithm, proposed in [15], was applied.

The data processing is followed by the classification stage. The classification model should recognize some user-specific patterns in the processed ECG signal and perform a matching with one of the corresponding classes (users). This is the last step of the identification process.

**Figure 1:**
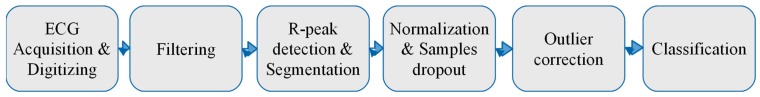
ECG-based identification process.

**Figure 2:**
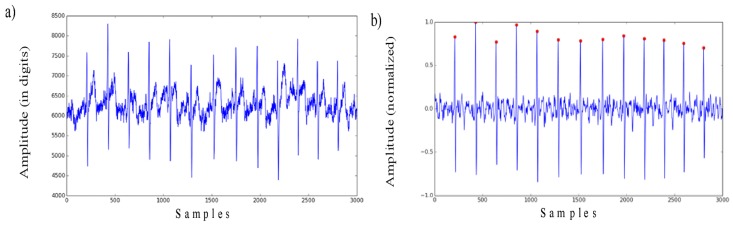
(**a**) Raw ECG signal and (**b**) ECG signal after filtering and normalization with the detected R peaks.

**Figure 3:**
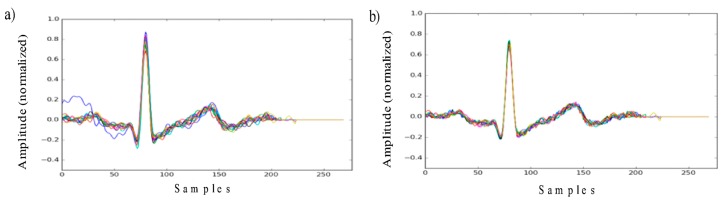
ECG segments (heart beats) aligned to the R peak (**a**) before and (**b**) after the outlier correction.

The entire ECG-based identification process is presented in Figure 1. The ECG signal waveforms appearing at different transformation stages are presented in Figure 2 and Figure 3.

## 3. Experimental Methodology

The whole idea of the article is to check whether the ECG signal is sustainable as a biometrics marker. To perform this task, the following methodology is proposed:

- First, different datasets from different sources (both self-collected and publicly available on the Internet) should be included into the study. It is expected that these datasets should have different origins and internal structures. The main parameters that should be taken into consideration are the number of users, total number of records, mean, minimum and maximum number of records per user, records length, etc. There are two constraints related to the dataset selection. The first one is that the dataset should contain records coming only from healthy people with a normal rhythm [16]. The second constraint assumes that the data should be recorded from the same scheme of electrode placement on the patient’s body, called the lead in cardiology. The reason for this is that the ECG waveform strongly varies when measured from different parts of the body.
- Second, the records classification should be performed through the use of different algorithms. In our experiments, we expect to use just one single heartbeat (the waveform between the onset of neighboring p-waves) for human identification. Thus, no sequential algorithm will be analysed here, only simple supervised machine learning techniques that map a multidimensional input vector (samples of an ECG heartbeat) onto a specified output vector (number of users). A comparison of various algorithms is required for two main reasons: first, to get some basic intuition on how some complex non-linear algorithms will behave, when compared to simpler linear algorithms while processing this kind of data; and second, to ensure that there is no bias in the different datasets. This means that the algorithms should demonstrate a similar behaviour on different datasets.
- Third, one of the most important stages while designing machine learning based experiments is to select the most appropriate metrics. In our case, since all datasets are relatively balanced, we decided to choose an identification accuracy (error rate) to estimate the algorithms’ performance.
- Finally, it is also proposed to have two-alternative data pre-processing algorithms in place. The first one is described in the section above. It means filtering, normalization and outlier correction. Another one proposes the use of a dimensionality reduction on the top. This trick is commonly used in machine learning and might help to improve the overall classification performance. In our case, it was decided to use PCA, as this is one of the simplest, most commonly used and efficient compression algorithms.

## 4. Datasets Description

Taking into consideration the requirements outlined above (see Section 3), the following four datasets were chosen for the current study, namely: Lviv Biometric Dataset (self-collected), Physionet ECG-ID dataset, Physionet QT dataset (only some records with a normal sinus rhythm) and Physionet MIT-BIH Normal Sinus Rhythm. A short description of each of these datasets, as well as a comparison of the main parameters, is presented below (see Table 1).

### 4.1. Lviv Biometric Dataset (LBDS)

This is a self-collected dataset available in [17]. All of the records were acquired using the eHealth Arduino extension board [14]. More details on the measurement procedure can be found in [8].

### 4.2. Physionet ECG-ID

This dataset was created for human identification purposes, as a part of the MSc thesis [18]. The records were acquired from 44 men and 46 women, between 13 to 75 years old [19]. For some users, there are only a few records available, which means that they were recorded for one day. For other users, there are over 20 records, collected periodically over a 6 months period. The Physionet ECG-ID database is available in [18].

### 4.3. Physionet QT-Database

This dataset was designed for the evaluation of the ECG heartbeat segmentation algorithms. It has annotations for each record, with boundaries of each heartbeat. This dataset includes not only records of healthy people, but also records of patients with cardiological disorders. Because of this, all annotations were manually reviewed in order to select records with a normal ECG rhythm. The following records were selected: sel103, sel117, sel123, sel16265, sel16272, sel16273, sel16420, sel16483, sel16539, sel16773, sel16786, sel17152, sel17453, sel301, sel302, sel306, sel307, sel310, sele0111, sele0124, sele0133, and sele0210. The Physionet QT database is available in [20,21].

### 4.4. Physionet MIT-BIH Normal Sinus Rhythm

This database includes ECG records obtained by the Arrhythmia Laboratory at Boston’s Beth Israel Hospital. The records originate from healthy people with no significant arrhythmias between 20 to 50 years old. The Physionet MIT-BIH Normal Sinus Rhythm database is available at [22].

## 5. Results and Discussion

Both the datasets selection and ECG signal pre-processing stages have already been described in the previous sections. The final stage is down to the user identification. It can be considered as a classification task, because the identification algorithm must match each ECG record to one of the existing users (classes). In general, the classification is done using machine learning techniques. The machine learning approach requires the selection of an appropriate algorithm that is powerful enough to model complex internal data relations and dataset splitting for a correct estimation of the classifier performance in real-world applications [23].

Machine learning algorithms have different natures, are based on different ideas and mathematical frameworks and are typically used in different applications. These factors should be taken into consideration when selecting the most suitable algorithm for ECG identification. The following seven algorithms have been chosen as the most promising: Logistic Regression, Support Vector Machine (SVM), Linear Discriminant Analysis (LDA), Naive Bayes, K Nearest Neighbor (KNN), Neural Networks or Multilayer Perceptron (MLP), Extreme Gradient Boosting (xGboost), Random Forest [24,25].

For multi-layer perceptron, the following configurations were used: 1 hidden layer with 50 neurons; 2 hidden layers with 50 and 30 neurons in each layer; and 3 hidden layers with 70, 50, and 30 neurons in each layer, respectively. The Rectified Linear Unit was selected as the activation function for the hidden layers and softmax as the activation function for the output layer. Training algorithm—RMSprop, number of training epochs—1000, learning rate—0.0001, batch size—100, loss function—categorical cross-entropy. For the other algorithms, we used the default configuration recommended by the sklearn framework (for example, in the case of PCA, the number of components was set to 30).

Dataset splitting requires their division into two subsets: a training and a test set. The samples from the training set are used to fit a classification model, while the samples from the test set are used to provide an unbiased evaluation of the model performance. The test set must be carefully prepared, as it should realistically represent the real-world data that the classification model would operate on.

As ECG-ID and LBDS have multiple records per user, we have split the test and the training set based on the records level. Some records will be randomly selected as the training set, while the remaining ones are included in the test set. Experiments will be conducted for the training and test set ratios of 0.7 and 0.3, respectively. To achieve a more realistic identification performance, the dataset split was done 5 times, after which the mean values for each subset was calculated.

For the MIT-BIH Normal Sinus rhythm and QT database, just one record per user is available. However, these records are of quite a substantial length. The idea is to use the time split for the training and the test set. In this case, the training and test ratios were also assumed to be 0.7 and 0.3.

Furthermore, as mentioned in Section 3, the classification models have been trained for two different scenarios: with and without PCA compression. The only exception here is neural networks, because they are complex non-linear models, which can learn efficient data compression in the first hidden layer. Thus, in this case it makes no sense to use PCA here. All results of our experiments are gathered in Table 2.

As one can see in Table 2, all of the algorithms seem to behave similarly across all of the datasets. Simple algorithms, like KNN and linear models (logistic regression, LDA, SVM), proved to work surprisingly well. Some other simple algorithms, like Naive Bayes, gradient boosting and random forest, performed relatively poorly. Neural networks also seem to guarantee a very high accuracy, which was pretty much expected, in view of their complex non-linear nature and modeling capacity. The PCA compression might slightly improve the accuracy for some datasets, while decreasing it for others. Consequently, it seems that there is no need to include PCA in the data preprocessing pipeline.

The best accuracy was achieved by LDA and MLP for all four datasets. KNN shows high results for all datasets, except for the MIT-BIH Normal Sinus Rhythm. Given that this database is much larger compared to the other ones, it is not clear whether KNN would scale well enough for a larger number of users and records. MLP and xGboost were the most time-consuming algorithms to train whilst logistic regression and LDA were among the fastest algorithms.

Another important observation, based on the results from Table 2, is that the hardware parameters (e.g., measurement instrumentation, lead type, and sampling rate) do not affect the identification results significantly. The lowest accuracy was achieved for the ECG-ID database (potentially because of the highly skewed classes and larger number of users) and the MIT-BIH Normal Sinus Rhythm (potentially difficult to scale on a much bigger number of samples).

## 6. Conclusions

The results we have obtained prove that the ECG signal is a valid biometric marker that is very robust to hardware variations, noise and artifacts presence, that is stable over time, and that is scalable over quite a solid number of users (>90). It is also hard to steal or mimic, is easy to measure, etc.

The biometric system allows for the achievement of a high operational speed, as just one heartbeat (average duration of less than 1 second) is enough to guarantee very good classification results (~90%). On the other hand, the outlier correction requires at least five heartbeats, which means that in a real-world application the overall response time will take at least 5 seconds.

The most promising algorithms for ECG identification are linear discriminant analysis (LDA), k-nearest neighbor (KNN), and neural networks (MLP). Another important conclusion clearly confirmed by our experiments is that PCA compression is not worth using at the data preprocessing stage, as in some cases it might reduce accuracy.

The following ideas might be interesting as potential future research topics: the estimation of system scalability for bigger datasets (e.g., mixed from different sources, and augmented using generative models), optimizing training hyperparameters for artificial neural networks, and performing a sequential analysis of neighboring heartbeats on the classification stage.

## Figures and Tables

**Table 1:** Basic parameters of the ECG datasets.

Parameter	LBDS	ECG-ID	QT	Normal Sinus Rhythm
Lead	modified I-lead (from the fingers of the right and left hand)	I-lead	I-lead	I-lead
Number of users	53	90	22	18
Total number of records	545	310	22	18
Records per user	from 3 to 15	from 1 to 22	1	1
Sampling rate	277 Hz	500 Hz	250 Hz	125 Hz
Average record time	~10 seconds	20 seconds	15 minutes	~10:20 hours (from 8:00 to 13:50 hours)

**Table 2:** ECG identification results.

	Physionet ECG-ID	LBDS	Physionet QT	MIT-BIH Normal Sinus Rhythm
Logistic Regression	0.8286	0.9417	0.8809	0.7492
SVM classifier	0.8817	0.9599	0.9174	0.7707
LDA classifier	0.9328	0.9831	0.9659	0.9017
KNN classifier	0.8903	0.9746	0.9686	0.7967
Naive Bayes	0.7003	0.9587	0.9034	0.6607
Random Forest	0.8362	0.9546	0.9278	0.8192
xgboost classifier	0.7352	0.9126	0.9191	0.8591
MLP (1 hidden layer)	0.8933	0.9711	0.9162	0.8925
MLP (2 hidden layer)	0.8976	0.9464	0.9478	0.8744
MLP (3 hidden layer)	0.8406	0.92373	0.9294	0.8808
PCA + Logistic Regression	0.8286	0.9383	0.8465	0.7335
PCA + SVM classifier	0.8865	0.9593	0.8832	0.7472
PCA + LDA classifier	0.9536	0.9833	0.9481	0.8798
PCA + KNN classifier	0.8913	0.9758	0.9675	0.7957
PCA + Naive Bayes	0.6211	0.9511	0.8915	0.6681
PCA + Random Forest	0.7782	0.9199	0.8947	0.7418
PCA + xgboost classifier	0.6723	0.8911	0.9460	0.7305

## Abbreviations

The following abbreviations are used in this manuscript:

ECG	Electrocardiogram
ADC	Analog to Digital Converter
MCU/PC	Microcontroller Unit/Personal Computer
PCA	Principle Component Analysis
LBDS	Lviv Biometric Dataset
SVM	Support Vector Machine
LDA	Linear Discriminant Analysis
KNN	K Nearest Neighbor
MLP	Multilayer Perceptron
